# microRNA profiling in the zoonotic parasite *Echinococcus canadensis* using a high-throughput approach

**DOI:** 10.1186/s13071-015-0686-8

**Published:** 2015-02-06

**Authors:** Natalia Macchiaroli, Marcela Cucher, Magdalena Zarowiecki, Lucas Maldonado, Laura Kamenetzky, Mara Cecilia Rosenzvit

**Affiliations:** Instituto de Investigaciones en Microbiología y Parasitología Médica (IMPaM), Facultad de Medicina, Universidad de Buenos Aires (UBA)-Consejo Nacional de Investigaciones Científicas y Tecnológicas (CONICET), Paraguay 2155, Piso 13, CP 1121 Buenos Aires, Argentina; Parasite Genomics, Wellcome Trust Sanger Institute, Wellcome Trust Genome Campus, Hinxton, Cambridge, CB10 1SA UK

**Keywords:** microRNAs, High-throughput, *Echinococcus canadensis*, Echinococcosis, Cestode, Parasite, Platyhelminth

## Abstract

**Background:**

microRNAs (miRNAs), a class of small non-coding RNAs, are key regulators of gene expression at post-transcriptional level and play essential roles in fundamental biological processes such as development and metabolism. The particular developmental and metabolic characteristics of cestode parasites highlight the importance of studying miRNA gene regulation in these organisms. Here, we perform a comprehensive analysis of miRNAs in the parasitic cestode *Echinococcus canadensis* G7, one of the causative agents of the neglected zoonotic disease cystic echinococcosis.

**Methods:**

Small RNA libraries from protoscoleces and cyst walls of *E. canadensis* G7 and protoscoleces of *E. granulosus sensu stricto* G1 were sequenced using Illumina technology. For miRNA prediction, miRDeep2 core algorithm was used. The output list of candidate precursors was manually curated to generate a high confidence set of miRNAs. Differential expression analysis of miRNAs between stages or species was estimated with DESeq. Expression levels of selected miRNAs were validated using poly-A RT-qPCR.

**Results:**

In this study we used a high-throughput approach and found transcriptional evidence of 37 miRNAs thus expanding the miRNA repertoire of *E. canadensis* G7. Differential expression analysis showed highly regulated miRNAs between life cycle stages, suggesting a role in maintaining the features of each developmental stage or in the regulation of developmental timing. In this work we characterize conserved and novel *Echinococcus* miRNAs which represent 30 unique miRNA families. Here we confirmed the remarkable loss of conserved miRNA families in *E. canadensis*, reflecting their low morphological complexity and high adaptation to parasitism.

**Conclusions:**

We performed the first in-depth study profiling of small RNAs in the zoonotic parasite *E. canadensis* G7. We found that miRNAs are the preponderant small RNA silencing molecules, suggesting that these small RNAs could be an essential mechanism of gene regulation in this species. We also identified both parasite specific and divergent miRNAs which are potential biomarkers of infection. This study will provide valuable information for better understanding of the complex biology of this parasite and could help to find new potential targets for therapy and/or diagnosis.

**Electronic supplementary material:**

The online version of this article (doi:10.1186/s13071-015-0686-8) contains supplementary material, which is available to authorized users.

## Background

The parasitic cestode *Echinococcus canadensis* is one of the causative agents of cystic echinococcosis, a chronic and disabling parasitic disease considered neglected by the World Health Organization. This disease is associated with poverty and poor hygiene practices, particularly in livestock-raising communities [1]. *E. canadensis* is a member of the complex *Echinococcus granulosus sensu lato* (s. l.) [2] and belongs to the class Cestoda, phylum Platyhelminths. *E. granulosus* s. l. life cycle involves two mammalian hosts. In the intermediate host, mainly ungulates and accidentally humans, the metacestode or hydatid cyst develops. The metacestode is a unilocular fluid-filled cyst circled by a cyst wall (CW) that consists of an inner germinal layer and an outer acellular laminated layer, and is surrounded by an adventitial layer from host-origin. The germinal layer produces small immature worms named protoscoleces (PS) which develop into adult strobilated worms in the gut of the definitive hosts, mostly canids. *Echinococcus* s. l. parasites display some unique characteristics such as the ability of the germinal layer to undergo practically unlimited asexual proliferation. Also, these parasites have a high degree of developmental plasticity which allows the PS to develop into an adult worm in the definitive host and to de-differentiate into secondary hydatid cysts if rupture and content leakage from the primary cyst occur within the intermediate host. *E. canadensis* is composed by 4 genotypes: *E. canadensis* G6-G8 and G10 [2]. Among them, *E. canadensis* G7 is highly adapted to pigs and wild boars and is able to infect humans [3,4]. It differs from other members of the complex in morphology, development and genetic traits [5], including genomic organization and abundance of repetitive DNA elements [6], composition and sequence of antigen-coding genes such as Antigen B [7,8] and the vaccine antigen EG95 [9] which was recently shown to differ in antigenicity with EG95 from other species of *E. granulosus* s. l. [10]. Recently, we have shown the inability of *E. canadensis* G7 protoscoleces to establish secondary hydatid cysts in mice [11], adding more evidence to the distinctiveness of this species. The particular developmental and metabolic properties of these cestode parasites highlight the importance of studying the underlying molecular basis. This could help, in turn, to find new control strategies by discovering essential and specific molecules which could be considered as potential targets for therapy and/or diagnosis.

microRNAs (miRNAs) are small ~22 nucleotides (nt) non-coding RNAs with a major role in regulation of gene expression that play critical roles in diverse cells and tissues during plant and animal development [12]. In the canonical biogenesis pathway, miRNAs are transcribed by RNA polymerase II into long primary miRNAs (pri-miRNAs) that are processed by the RNAse III enzyme Drosha to produce a ~70 nt long stem-loop miRNA precursor (pre-miRNA) which is further processed by another RNAse III enzyme, Dicer, into a miRNA-miRNA* duplex. One of the two strands of this duplex, the mature miRNA, loads into a microRNA Induced Silencing Complex (miRISC) and guides Argonaute (AGO) proteins to complementary mRNA sequences to repress their expression. The other strand, known as the star miRNA (miRNA*), has typically been assumed to be a carrier strand. The major determinant of AGO binding to the mRNA is a 6–7 nt sequence at the 5’ end of the mature miRNA known as the “seed region” [13]. miRNAs down-regulate gene expression post-transcriptionally by binding to the mRNA of their target genes and promoting their cleavage, or more commonly in metazoans, their translational repression and/or destabilization. The importance of miRNAs in key biological processes such as development, cell proliferation, cell differentiation and metabolism has been widely documented since their discovery [14]. To gain an understanding of the role of miRNAs in the regulation of developmental and metabolic processes in *Echinococcus*, we have performed high-throughput identification and profiling of miRNAs in different life cycle stages of *E. canadensis* G7. In a previous work, we have shown the presence of miRNAs by using a low scale cloning and sequencing approach of small RNAs from *E. canadensis* G7 protoscoleces [15]. However, the knowledge of *E. canadensis* miRNA expression profile is still limited. The aim of this study is to perform a comprehensive comparative analysis of miRNAs in *E. canadensis* G7. An in depth identification and expression analysis of these molecules will allow the study of their role/s in parasite biology and will provide novel targets for tapeworm control.

## Methods

### Parasite material

Fertile hydatid cysts were obtained from the livers of naturally infected swine and sheep provided by abattoirs from Buenos Aires and Rio Negro provinces, Argentina. The animals involved in this study were not subjected to any experimental procedure. All the samples for the study were collected post-mortem in commercial abattoirs. Two cysts (N = 2) were obtained in order to have biological replicates. The hydatid fluid was aseptically aspirated from cysts with a syringe. Protoscoleces (PS) were recovered from aspirated fluid and extensively washed in PBS to remove dead protoscoleces and cyst wall debris, as described [16]. Then, the hydatid cyst wall (CW) (germinal and laminated layers) was carefully recovered from cyst with forceps and extensively washed in PBS to remove host cells and protoscoleces. Cyst wall samples were observed under a light microscope to verify the absence of protoscoleces. One fraction of freshly isolated PS from each cyst was used to determine viability by eosine exclusion test. Samples showing more than 90% viability were frozen in liquid nitrogen and stored at −80°C until RNA extraction. The species and genotype were determined by sequencing a fragment of the mitochondrial cytochrome c oxidase subunit 1 (CO1), as previously described [15]. The resulting species and genotype were *E. canadensis* G7 and *E. granulosus* s. s. G1 for samples from swine and sheep, respectively.

### Small RNA isolation

RNA enriched in small RNAs (<200 nt) were purified from protoscoleces and cyst walls using mirVana miRNA Isolation Kit (Ambion) according to the manufacturer’s instructions. In the case of cyst wall samples, an additional centrifugation step at 12,000 g for 10 min at 4°C was performed after sample disruption in lysis solution in order to remove insoluble material of the laminated layer. RNA was then precipitated overnight at −20°C with 0.1 volumes of 3 M sodium acetate (pH 5.2), 2.5 volumes of 100% ethanol and glycogen. RNA was centrifuged at 14,000 g for 30 min at 4°C, washed in 80% ethanol, air dried at room temperature and resuspended in nuclease-free water. Samples were stored at −80°C until cDNA library construction. RNA concentration was determined using a Qubit Fluorometer (Invitrogen) and RNA integrity was assessed using an Agilent 2100 Bioanalyzer according to the manufacturer’s protocol*.*

### Small RNA library construction and sequencing

A NEBNext Small RNA Library Prep Set for Illumina (NEB) was used to prepare the libraries following the instruction’s manual. For each small RNA library construction, 1.5 μg of RNA enriched in small RNAs (<200 nt) was used as starting material. For each sample type; CW from *E. canadensis* G7 (CWG7), PS from *E. canadensis* G7 (PSG7), PS from *E. granulosus* s. s. G1 (PSG1); two libraries were constructed from two independent samples in order to count with biological replicates. After adaptors ligation, reverse transcription and PCR amplification were performed. Then, the libraries were size selected: two bands centering at 140 bp and 150 bp which corresponded to constructs derived from RNA fragments of sizes around 21 and 30 nucleotides, respectively, were isolated from a 6% polyacrilamide gel. Then, the size selected libraries were validated with an Agilent 2100 Bioanalyzer to check size and purity. The concentration of each cDNA library was determined using a Qubit Fluorometer (Invitrogen) and samples were diluted for direct sequencing using an Illumina cBot and Genome Analizer IIx sequencing platform at the Molecular Biology Unit of Institut Pasteur de Montevideo, Uruguay. All six libraries were constructed in parallel and sequenced in the same lane for 72 cycles.

### Source of genome assemblies and annotations

The high quality *Echinococcus multilocularis* genome assembly version 4 and *E. granulosus* s. s. G1 draft genome assembly [17], were obtained from the Sanger Institute FTP site [18]. The *Echinococcus* genome annotation (CDS, tRNA, rRNA) was obtained from GeneDB website [19]. Additional rRNA sequences from flatworms [20] and flatworms DNA repetitive elements were downloaded from NCBI [21]. *E. granulosus* s. s. G1 long non-coding RNAs (lncRNAs) [22] were retrieved from PartiGeneDB website [23]. *Echinococcus* hairpin sequences were obtained from miRBase 20. All annotated sequences, along with novel miRNA precursor sequences identified in this study, were used to construct an in-house database for small RNA library data classification.

### Bioinformatics analysis of *Echinococcus* small RNAs

Illumina raw sequence reads produced by deep sequencing were preprocessed using FASTX-Toolkit [24] before mapping to reference genome. After adapter trimming, low quality reads and reads shorter than 18 nt were removed to obtain clean reads. Then, identical clean reads were collapsed into unique sequences with associated read counts. To classify all small RNA library sequences as miRNAs, rRNA, tRNA, CDS/sense CDS/antisense, lncRNAs and repeats, the processed reads were first mapped to *E. multilocularis* reference genome (version 4) with Bowtie (version 0.12.7) [25] with the option -v 2 that reports read mappings with up to two mismatches. All mapped reads were then analyzed by BLASTN (e-value 0.01) against an in-house database that included all miRNAs identified in this study (as described in “miRNA identification” section) and classified into the above mentioned categories. Reads with no match were grouped into “unknown” category. Length distribution analysis of total mapped reads and total miRNA reads was performed.

### miRNA identification

**Figure 1:**
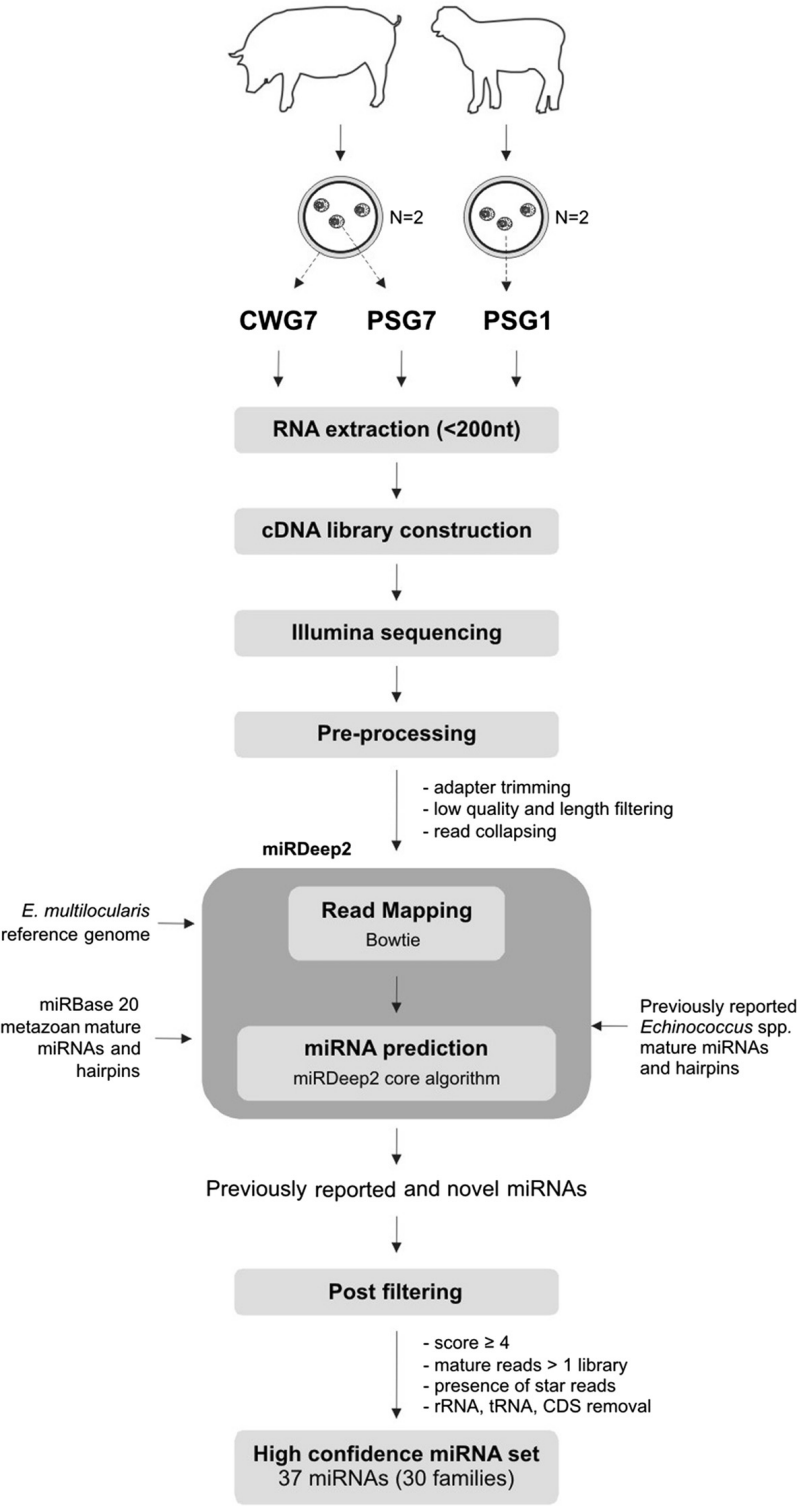
**Experimental and bioinformatic workflow for** ***Echinococcus canadensis*** **G7 and** ***Echinococcus granulosus sensu stricto*** **G1 miRNA identification.** CWG7: *E. canadensis* G7 cyst wall, PSG7: *E. canadensis* G7 protoscoleces, PSG1: *E. granulosus* s. s. G1 protoscoleces. Two biological replicates for each sample type were used.

To identify previously reported and novel miRNAs from the small RNA libraries, the miRDeep2 software package [26] was used. The unique sequences were mapped to *E. multilocularis* reference genome with the read aligner Bowtie (mapper module) using default parameters, allowing only alignments with 0 mismatches in the first 18 nt of a read sequence and up to two mismatches after nt 18, and keeping only reads that did not map more than five times to the genome. For miRNA prediction with the core algorithm of miRDeep2, all metazoan mature miRNAs and hairpins including previously reported *Echinococcus* mature miRNAs and hairpins (both retrieved from miRBase release 20) along with mapped reads from the previous step were used as input (Figure 1). The initial miRDeep2 output list of candidate miRNA precursors of each library was manually curated to generate a final high confidence set of miRNAs retaining only candidate novel precursors with i) miRDeep2 score ≥ 4 ii) significant randfold p-value < 0.05 iii) mature reads in more than one library and iv) presence of star strand. The candidate novel precursor sequences were then analyzed using BLASTN (e-value 0.01) against sets of rRNAs, tRNAs, CDS, lncRNAs and repeats. Predictions that overlapped with these categories were removed. The final set of candidate *E. multilocularis* hairpins was BLAST searched against *E. granulosus* s. s. G1 draft genome assembly to obtain the corresponding hairpin precursors. Nucleotide sequence data reported in this paper have been submitted to the miRBase database.

### miRNA annotation, identification of families and conservation analysis

To identify homologous miRNAs in *Echinococcus*, the full-length mature miRNA sequences were compared to previously reported miRNAs present in miRBase 20 using SSEARCH [27] (e-value cutoff of 100) allowing only sense matches and applying a 70% nucleotide identity cut-off and a seed match criteria: identical nucleotides 1–7 or 2–8 from the 5’ end of the mature miRNA. These criteria have been used in a recent miRNA study [28] for gene name assignment. Those miRNAs that did not meet the above mentioned requirements were considered novel candidate miRNAs. To identify miRNA families within *Echinococcus*, all-against-all pairwise sequence alignments were computed using BLAST and all sequences sharing the seed region (nt 1–7 or nt 2–8) were considered to belong to the same *Echinococcus* miRNA family. To analyze conservation of *Echinococcus* miRNA families, mature miRNA sequences were compared to those previously reported present in miRBase 20 for selected phyla; Cnidaria, Nematoda, Arthropoda, Annelida and the subphylum Vertebrata, using only a seed match criteria. For sequence conservation analysis, the full-length mature sequences of selected *Echinococcus* miRNA families identified in this study were aligned against a set of homologous full-length mature sequences of three selected model species: *Homo sapiens* (Chordata), *Caenorhabditis elegans* (Nematoda)*, Drosophila melanogaster* (Arthropoda) and two platyhelminths: *Schmidtea mediterranea* (Turbellaria) and *Schistosoma japonicum* (Neodermata) using the multiple sequence alignment tool ClustalX [29].

### miRNA abundance and differential expression analysis

For analysis of miRNA abundance levels, read counts of each individual miRNA in a sample were normalized to the total number of mature miRNA read counts in that sample according to [30]. Then, normalized miRNA read counts from biological replicates were averaged. A correlation analysis between independent biological replicates from each sample type was performed. For this purpose, miRNA read counts in a replicate were plotted against miRNA read counts in the other replicate. All miRNAs identified in this study where considered for this analysis. Differential expression analysis of miRNAs between stages or species was performed by DESeq using raw reads as input [31]. miRNAs expressed in both stages/species that showed −1 ≥ log_2_ fold change ≥ 1 and p-adjusted <0.001 were considered differentially expressed.

### Analysis of miRNA expression by poly-A RT-qPCR

Poly-A RT-qPCR [32] was performed to validate miRNA expression of 7 randomly selected diferentially expressed miRNAs between PSG7 and CWG7 identified by DESeq from deep sequencing data. Relative quantification between PSG7 and CWG7 was performed using the 2^-∆∆CT^ method [33] using miR-71-5p as endogenous control since it was found to be consistently expressed in both sample types. Reactions for PSG7 and CWG7 for each gene and endogenous control were performed in the same run to avoid variation in amplification conditions between runs. Three biological replicates were used in order to estimate the significance of the observed differences. Statistical significance was assessed by performing a Student’s *t*-test. miRNAs showing −1 ≥ log_2_ fold change ≥ 1 and p-value < 0.05 were considered differentially expressed. Furthermore, the expression levels of 8 additional randomly selected miRNAs were also validated in PSG7 and CWG7 samples by poly-A RT-qPCR. The quantification of each miRNA relative to miR-71-5p in PSG7 and CWG7 samples was calculated using the equation 2^-∆Ct^; ∆Ct = Ct_miRNA_-Ct_reference_. Prior to the reverse transcription reaction, 1 μg of the small RNA fraction was treated with DNase I (Invitrogen) according to the protocol of the manufacturer and then polyadenylated with *E. coli* Poly(A) Polymerase (NEB) for 60 min at 37°C in a 20 μl reaction volumen. cDNA was synthesized from 100 ng of polyadenylated small RNAs from either PSG7 or CWG7 using SuperScript III Reverse Transcriptase (Invitrogen) in a 20 μl reaction volumen. Controls without reverse transcriptase were included for each sample. Reverse transcription was performed by using the following program: 60 min at 50°C, 15 min at 70°C. For each PCR, 5 μl of diluted cDNA (1:100) was mixed with 0.5 μl of each primer (10 μM), 4 μl 5× HOT FIREPol® EvaGreen® qPCR Mix Plus (Solis BioDyne) and 10 μl sterile water in a final volumen of 20 μl. Real time quantitative PCR was performed using an ABI Prism 7500 Real-Time PCR system (Applied Biosystems, Foster City, USA). Cycling conditions were: 95°C for 15 min, followed by 40 cycles of 95°C for 15, 60°C for 20 s and 72°C for 32 s. Dissociation curve analysis was carried out at the end of each PCR run to verify amplification specificity for each gene. The baseline and Cq were automatically determined using 7500 System version 1.3.0 (Applied Biosystems). No template controls were included for each primer pair and each qPCR reaction was carried out in duplicate. Ten-fold dilution series were performed with pooled cDNA from all samples tested in this study to construct standard curves for each primer pair. The mean Cq values for each serial dilution were plotted against the logarithm of the cDNA dilution factor. The amplification efficiency for each miRNA was calculated from the expression [10^(−1/S)^-1] × 100%, where S represents the slope of the linear regression. The primer sequences and their PCR efficiencies are listed in Additional file 1.

### Data access

The small RNAseq data from this study have been deposited in NCBI’s Gene Expression Omnibus [34] and are accessible through GEO Series accession number GSE64705 (http://www.ncbi.nlm.nih.gov/geo/query/acc.cgi?acc=GSE64705).

## Results

### Small RNA library sequence analysis

**Figure 2:**
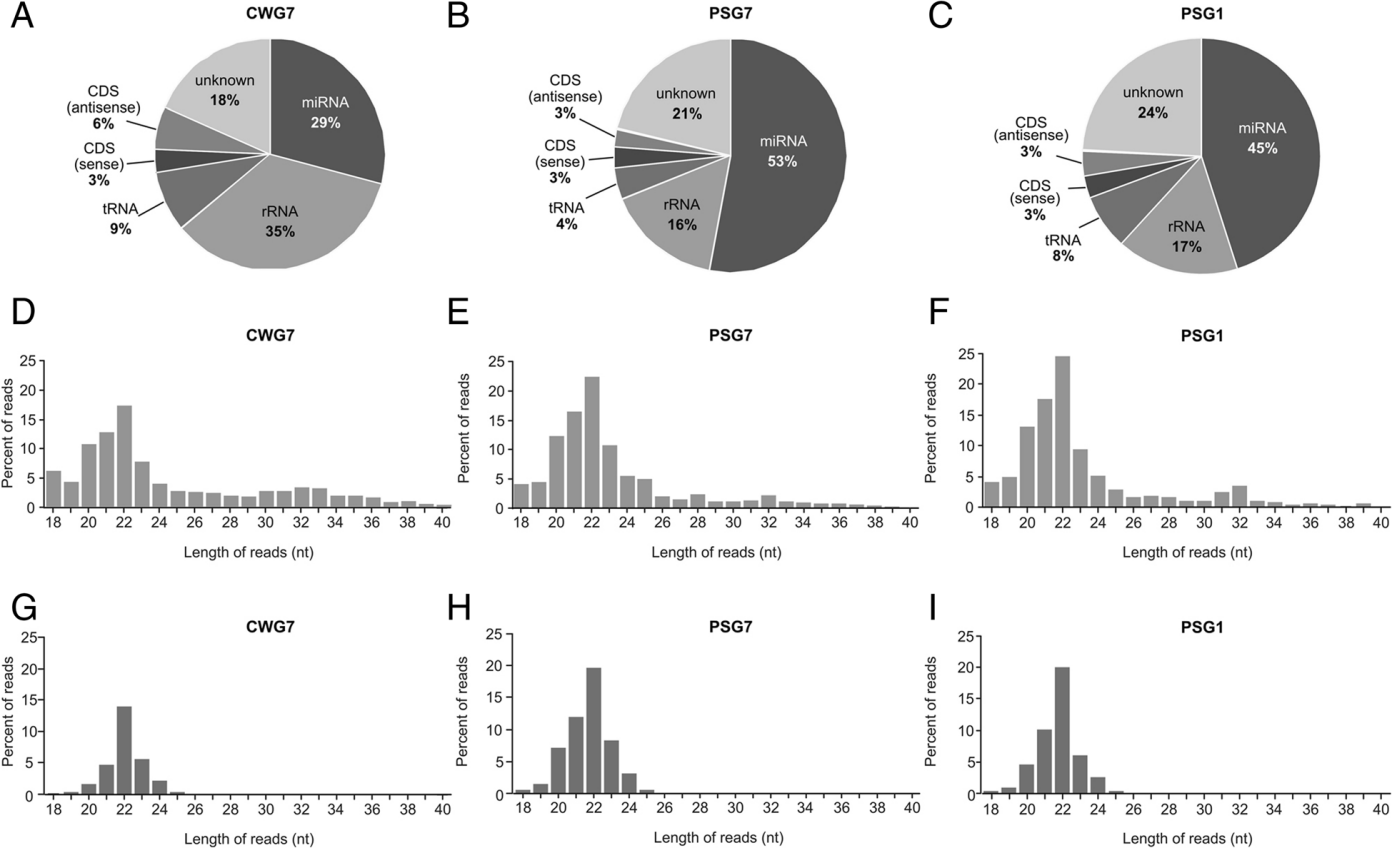
**Small RNA library composition and length profiles of** ***Echinococcus canadensis*** **G7 and** ***Echinococcus granulosus sensu stricto*** **G1 sequencing data. A-C)** The average proportion of reads representing different categories of small RNAs relative to the total number of mapped reads in each library is shown for each sample type. Reads matching lncRNAs and repeats are not represented due to their low relative abundance (<0.2%). **D-F)** Length distribution of total mapped reads. **G-I)** Length distribution of miRNA reads. CWG7: *E. canadensis* G7 cyst wall. PSG7: *E. canadensis* G7 protoscoleces. PSG1: *E. granulosus s. s.* G1 protoscoleces.

In order to explore the whole repertoire of miRNAs expressed by *E. canadensis* G7 and compare miRNA expression between stages, small RNA libraries from cyst walls (CWG7) and PS (PSG7) from *E. canadensis* G7 were sequenced using Illumina technology. In addition, a sample of *Echinococcus granulosus* s. s. G1 (PSG1) was included in order to compare the miRNA repertoire with our species of interest. Library construction and analysis was performed using the same methodology as for *E. canadensis* G7 samples. To count with biological replicates, two libraries were constructed from independent samples of each type, totaling six libraries. The experimental and bioinformatic workflow developed for this study is shown in Figure 1. Illumina deep sequencing produced between ~ 2.0 and 5.3 million raw reads per sample (Table 1) from which ~ 1 to 3.2 millons mapped to the reference genome, the high quality 115 Mb *E. multilocularis* version 4 [17]. The collapsed (unique) reads ranged from 129,557 to 340,876. We obtained a high percentage of genome mapping, from 46% to 88% depending on sample type. The proportion of sequences that mapped to the genome was lower for CW than for PS samples (Table 1), probably due to host origin material in CW that is in close contact with the host adventitial layer that surrounds the metacestode. Next, we analyzed the composition of the small RNA reads in each library. Mapped reads were classified as miRNAs, tRNA and rRNA derived sequences, sequences mapping in sense (CDS/sense) and in antisense (CDS/antisense) orientation to CDS, lncRNAs and repeats. Sequences that could not be classified in any category were grouped as “Unknown”. The proportion of reads assigned to each category for CWG7, PSG7 and PSG1 libraries can be observed in Figure 2A-C. miRNA reads were the most abundant category in PSG7 and PSG1 libraries accounting for 53% and 45% of total mapped reads, respectively. The percentage of miRNA reads in CWG7 was lower (29%) than in protoscoleces. A higher proportion of rRNA was observed in CW samples (35%). Reads classified as tRNAs accounted for 4-9%. There was a low proportion of reads mapping to CDS (3-6%) and very few sequences classified as lncRNAs and repeats (Figure 2A-C, Additional file 2). Length distribution analysis showed that the small RNA profile of total mapped reads was similar in all samples and revealed one single prominent peak at 22 nt which is compatible with miRNAs (Figure 2D-F). The presence of miRNAs in all the libraries was further supported by the length distribution profile of reads from miRNA category (Figure 2G-I). No peak compatible with piRNAs (~30 nt) was observed in any sample (Figure 2D-F) which suggests that piRNAs are not expressed either in the metacestode or the protoscolex stages. Interestingly, when we analyzed the length distribution of reads that did not map to the parasite reference genome we found a peak at 31 nt in the CW libraries, which represents the parasite larval stage in direct contact with the intermediate host. This peak was absent in PS libraries. BLAST analysis of the 5 most abundant 31 nt sequences not-mapping to the parasite’s genome, corresponded to 5’ half-tRNAs from host origin (Additional file 3).

**Table 1:** **Summary of sequenced** ***Echinococcus canadensis*** **G7 and** ***Echinococcus granulosus sensu stricto*** **G1 small RNA libraries**

**Sample type** ^**a**^	**Biological replicate**	**Raw reads**	**Clean reads**	**Number of mapped reads**	**Number of unique mapped reads**	**Percentage of mapped reads (%)**
CWG7	1	3478621	2487372	1296835	181581	52.13
CWG7	2	2595548	2117367	964013	129557	45.52
PSG7	1	5336595	4065356	3292446	340876	80.98
PSG7	2	2596358	1882945	1655705	144788	87.93
PSG1	1	1952674	1642112	1302664	157098	79.32
PSG1	2	3108663	1956161	1674196	202776	85.58
Total		19068459	14151313	10185859	1156676	71.91^b^

^a^CWG7: *E. canadensis* G7 cyst wall, PSG7: *E. canadensis* G7 protoscoleces, PSG1: *E. granulosus* s. s. G1 protoscoleces.

^b^Average percentage of mapped reads from all samples.

### Identification of *E. canadensis* G7 miRNAs

**Figure 3:**
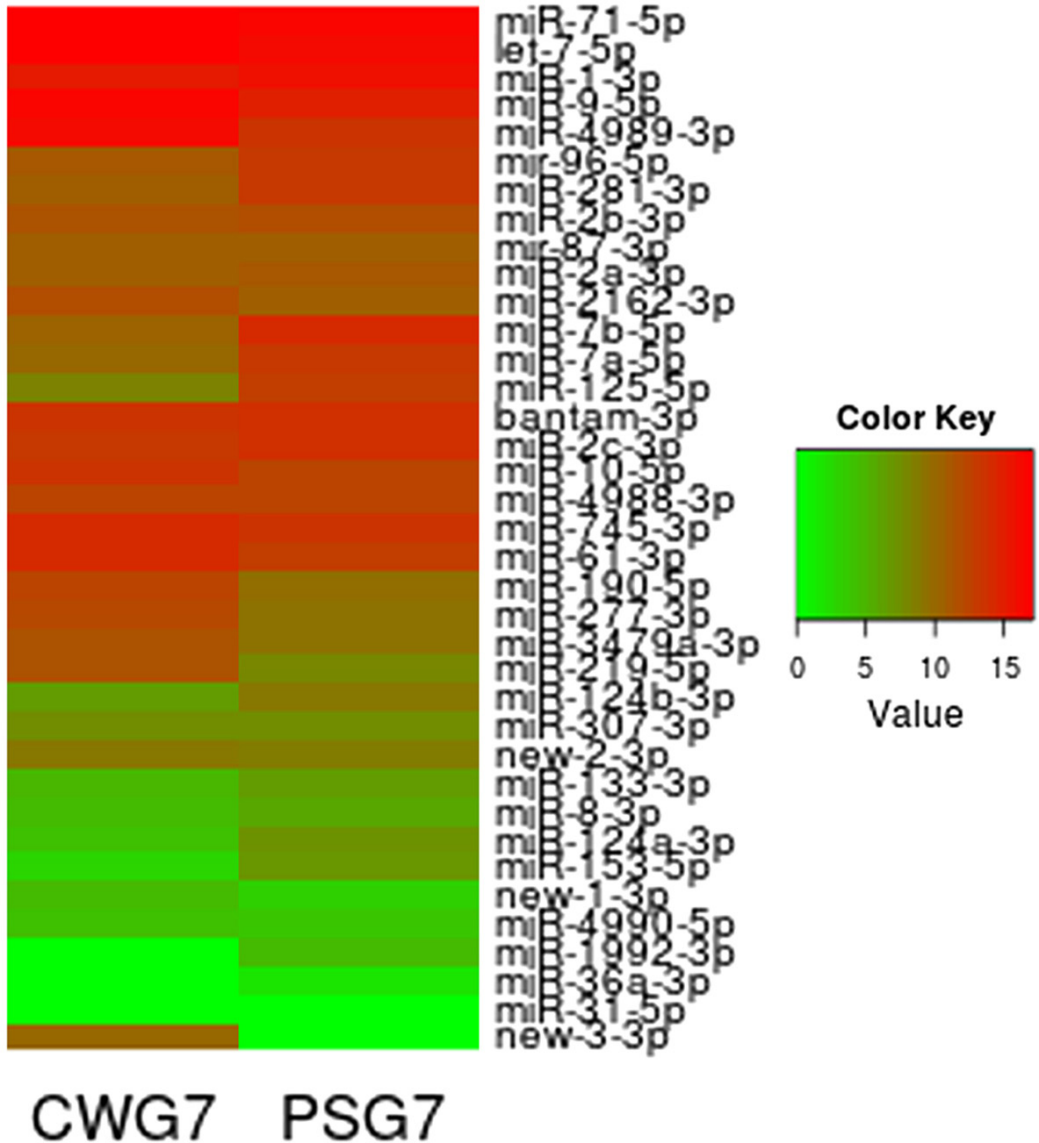
**Expression profile of the full miRNA repertoire of** ***Echinococcus canadensis*** **G7 cyst wall (CW) and protoscoleces (PS) samples.** Heatmap of log_2_-transformed normalized miRNA reads organized by their transcriptional abundance. miRNA expression is displayed using a color key where green corresponds to low and red to high numbers of miRNA normalized reads. (This figure appears in colour on the web).

The miRNA prediction program miRDeep2 [26] was used to identify miRNA genes from each small RNA deep-sequencing dataset. This algorithm has been used previously for miRNA identification in platyhelminths and nematodes [26,28,35-37] allowing the identification of conserved and novel miRNAs in several species. The pipeline used in this work produced a final high confidence set of 37 miRNAs: 32 conserved and 5 novel candidate miRNAs, representing in total 37 miRNA loci. Twenty three of the *E. canadensis* G7 identified miRNAs were previously reported by us [15]. Thus, 14 *E. canadensis* miRNAs were newly identified in this study. The conserved and candidate novel mature *E. canadensis* G7 and *E. granulosus* s. s. G1 miRNAs identified in this work are shown in Table 2. Three conserved and highly abundant miRNAs with particularly long hairpins: mir-87 (94 nt), mir-96 (97 nt), and mir-7b (97 nt) could only be detected after changing the miRDeep2 algorithmic parameters for the excision of hairpins as was previously described [36]. Expression of miR-96 was confirmed by RT-qPCR (Additional file 4). Star sequences were identified for all pre-miRNAs with the only exception of mir-36, adding confidence to the predictions obtained. Expression profiles based on normalized read counts of the full repertoire of *E. canadensis* G7 miRNAs identified in thiswork study are shown in Figure 3 and Additional file 5. Precursor sequences and raw read numbers of the full repertoire of *E. canadensis* G7 and *E. granulosus s. s.* G1 miRNAs identified in this study are shown in Additional file 6.

**Table 2:** **Conserved and candidate novel mature** ***Echinococcus canadensis*** **G7 and** ***Echinococcus granulosus sensu stricto*** **G1 miRNAs identified in this study**

**Identified microRNAs**	**Number**	**Mature miRNA name**
Conserved miRNAs	32	bantam-3p, let-7-5p, miR-1-3p, miR-2a-3p, miR-2b-3p, miR-2c-3p, miR-7a-5p, miR-7b-5p, miR-8-3p, miR-9-5p, miR-10-5p, miR-31-5p^a^, miR-36a-3p, miR-61-3p, miR-71-5p, miR-87-3p, miR-96-5p, miR-124a-3p, miR-124b-3p, miR-125-5p, miR-133-3p, miR-153-5p, miR-184-3p (former miR-4988), miR-190-5p, miR-219-5p, miR-277-3p, miR-281-3p, miR-307-3p, miR-745-3p, miR-1992-3p, miR-2162-3p, miR-3479a-3p
Candidate novel miRNAs	5	miR-4989-3p, miR-4990-5p, miR-new-1-3p, miR-new-2-3p, miR-new-3-3p^b^
Total number of miRNAs	37	

^a^Expression of miR-31-5p was only detected in *Echinococcus granulosus sensu stricto* G1.

^b^Expression of miR-new-3-3p was only detected in *Echinococcus canadensis* G7.

A dominant mature miRNA can be processed either from the 5’ or 3’ arm of the corresponding pre-miRNA. Here we found that in *E. canadensis* G7 and *E. granulosus s. s.* G1 most mature miRNAs (60%) are processed from the 3’ arm of the hairpin (Additional file 6). This slight bias toward 3’ arm usage was also observed in nematodes [38].

In this work, 21 from the 23 precursor sequences previously identified from *E. canadensis* G7 protoscolex stage [15] were successfully identified while two were not classified as miRNAs by the miRDeep2 algorithm. One of them, miR-4991, was ruled out since the relative position of the reads in the predicted precursor sequence was not compatible with miRNA biogenesis. The other, miR-4990, was classified as a valid hairpin using CID-miRNA [39] and was included in our miRNA set. Two miRNA clusters have been described in *Echinococcus,* with miRNAs in each cluster contained in a genomic region of <300 nt [15]. We investigated the genomic arrangement of novel miRNA genes identified in this study and found that mir-133 is located approximately 12 kb from mir-1. Although miR-1 and miR-133 clustering is highly conserved across metazoan species [40] it would be interesting to determine if both miRNAs form part of a single transcriptional unit in *E. canadensis* G7.

In this study, we showed that 6 miRNA families: mir-36, mir-92 (miR-3479 and miR-new-3), mir-67 (miR-307), mir-184, mir-281, mir-1992 which were considered lost in *Echinococcus* [36] are also present in *E. canadensis* G7. Additionally, we confirmed the expression of six miRNAs that were recently predicted from genome data: miR-31, miR-61, miR-133, miR-281, miR-2162 and bantam in *E. granulosus* s. l. [41]. The results of our work also showed the expression of miRNAs that were not identified by the bioinformatics approach such as miR-36, miR-307, miR-1992, mir-3479 [41], highlighting the potential of the high-throughput technology used in this study for miRNA discovery. Furthermore, the expression of miR-3479, miR-new-3, miR-184, miR-61, miR-281 and bantam was confirmed by RT-qPCR in CWG7 and PSG7 samples (Additional file 4).

### Highly expressed miRNAs in *E. canadensis* G7

**Figure 4:**
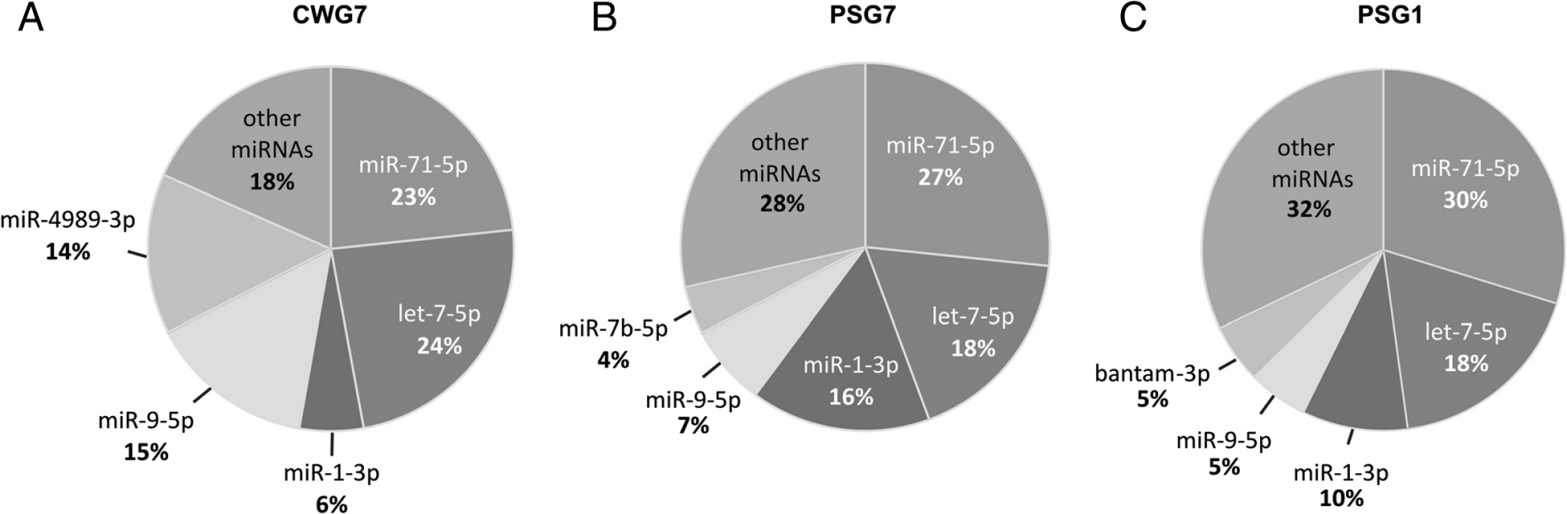
**Top five most abundant miRNAs in** ***Echinococcus canadensis*** **G7 and** ***Echinococcus granulosus sensu stricto*** **G1 datasets.** The average proportion of top five most abundant miRNA reads relative to the total number of mature miRNAs in each library, is shown for each sample type. **A)** CWG7: *E. canadensis* G7 cyst wall. **B)** PSG7: *E. canadensis* G7 protoscoleces. **C)** PSG1: *E. granulosus* s. s. G1 protoscoleces.

Since RNA-Seq has been shown to be highly accurate for quantifying expression levels, we considered the relative number of sequence reads of each miRNA as an indication of its abundance [42,43] in each sample. The top five most abundant miRNAs expressed in *Echinococcus* samples are shown in Figure 4. Interestingly, two miRNAs, miR-71 and let-7, were the most abundantly expressed miRNAs in all the analyzed samples, accounting for about 50% of the total miRNA expression in each sample, suggesting that these miRNAs are essential for *Echinococcus* survival in the intermediate host. Other highly expressed miRNA in all *Echinococcus* samples was miR-9. Interestingly, miR-4989 was one of the most expressed miRNAs in CW samples only. Finally, it was observed that the top five most abundant miRNAs accounted for 82% (CWG7), 72% (PSG7) and 68% (PSG1), of the total miRNA reads in each sample. Expression levels of highly expressed miRNAs (miR-71, miR-9, let-7 and miR-4989) were further validated by RT-qPCR in CWG7 and PSG7 samples (Additional file 4).

### Differential expression analysis

**Figure 5:**
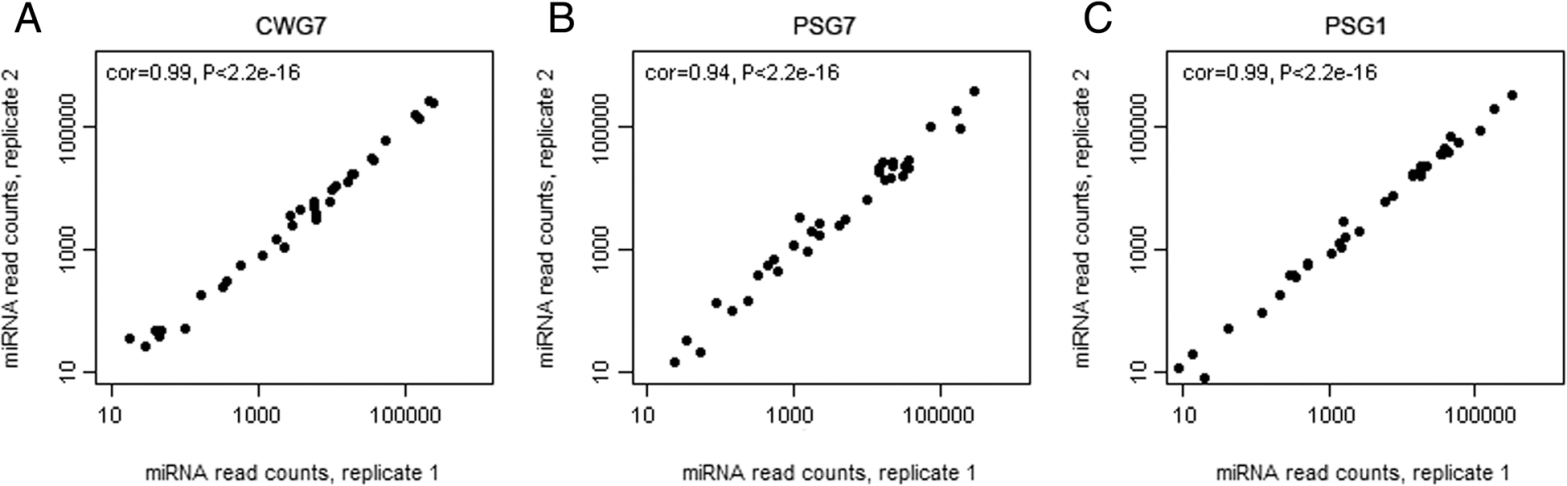
**Correlation analysis between independent biological replicates from each sample type of** ***Echinococcus canadensis*** **G7 and** ***Echinococcus granulosus sensu stricto*** **G1.** Each data point represents one miRNA. Pearson’s correlation coefficient and p-values are shown in each plot. **A)** CWG7: *E. canadensis* G7 cyst wall. **B)** PSG7: *E. canadensis* G7 protoscoleces. **C)** PSG1: *E. granulosus* s. s. G1 protoscoleces.

**Figure 6:**
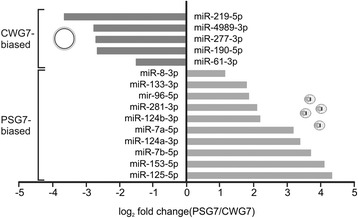
**Differentially expressed miRNAs between** ***Echinococcus canadensis*** **G7 cyst wall (CW) and protoscoleces (PS).** miRNAs that were expressed in both stages but showed −1 ≥ log_2_ fold change ≥1 and p-adjusted <0.001 are shown. Differential expression analysis performed by DESeq.

Since miRNA expression pattern throughout the development of an organism can help to predict their biological functions, differential expression analyses were conducted between two developmental stages of *E. canadensis* G7 and between protoscoleces from *E. canadensis* G7 and *E. granulosus* s. s. G1. First, we performed a correlation analysis between the independent biological replicates from each sample type which indicated high reproducibility (Figure 5) thus allowing differential expression analysis. Differential expression of all identified miRNAs between two conditions (stages or species) was analyzed using DESeq [31]. This approach has been used in other miRNA studies such as [44] and [45]. We found 15 miRNAs (43%) differentially expressed between PS and CW from *E. canadensis* G7 (Figure 6), suggesting that miRNAs could be involved in maintaining stage-specific features. Two of the five miRNAs up-regulated in CW, miR-4989 and miR-277, have the same seed region suggesting that they could share a set of targeted genes important for maintaining germinal layer features. Ten miRNAs are up-regulated in PS, a stage showing higher morphological organization and complexity. The most highly up-regulated miRNA in PS is miR-125. This miRNA is homologous to *C. elegans* lin-4, the first discovered miRNA that controls the developmental timing of the worm [46]. Differential expression of four selected up-regulated miRNAs in CWG7 samples (miR-4989, miR-277, miR-190, miR-61) and three selected up-regultated miRNAs in PSG7 samples (miR-125, miR-7a and miR-96) were validated by RT-qPCR (Table 3). A high correlation of log_2_ fold changes between RNAseq and RT-qPCR was found (correlation coefficient = 0.97).

**Table 3:** **Real time PCR validation of selected stage-biased expressed microRNAs in** ***Echinococcus canadensis*** **G7**

	**miRNA**	**RNAseq**	**p-adj** ^**1**^	**qPCR**	**p-value** ^**2**^
		**log**_**2**_ **FC**		**log**_**2**_ **FC** ^**3**^	
**CW-biased**	miR-4989-3p	−2,76	<0,001	−2,62	<0,01
	miR-277-3p	−2,70	<0,001	−2,98	<0,05
	miR-190-5p	−2,66	<0,001	−1,35	<0,05
	miR-61-3p	−1,50	<0,001	−1,34	<0,001
**PS-biased**	mir-96-5p	1,87	<0,001	1,19	<0,01
	miR-7a-5p	3,20	<0,001	2,74	< 0,001
	miR-125-5p	4,35	<0,001	2,63	< 0,001

^1^P-adjusted values according to DESeq.

^2^P-values based on t-test.

^3^Correlation coefficient of log2 fold changes between RNAseq and RT-qPCR = 0.97.

No significant differences were found in miRNA expression between protoscoleces from *E. canadensis* G7 and *E. granulosus* s. s. G1 in the present study.

### Conservation analysis of *E. canadensis* miRNA families

In order to determine the existence of *Echinococcus-*specific miRNA families, we characterized the mature sequences of the 37 miRNA loci identified in this study by searching miRBase v20 using only a seed match criterion. The evolutionary origin of *Echinococcus* (phylum Platyhelminths) miRNAs and their seed conservation in other phyla: Cnidaria, Nematoda, Arthropoda, Annelida and the subphylum Vertebrata are shown in Table 4. We found 28 *Echinococcus* conserved seed families, thus confirming the previously proposed loss of miRNA families in *Echinococcus* [36]. However, we observed that the percentage of miRNAs that have been lost is 39%, somewhat lower than the 50% inferred by Fromm et al., 2013 [36]. This difference is due to the finding of 6 miRNA families, mir-36, mir-92, mir-67, mir-184, mir-281, mir-1992 (Table 4), which in that study were considered lost in *Echinococcus*. Among the conserved families, we found that five of them have multiple members: miR-2 (miR-2a, miR-2b, miR-2c), miR-124 (miR-124a, miR-124b), miR-277 (miR-277, miR-4989, miR-new-2), miR-92 (miR-3479, miR-new-3) and miR-7 (miR-7a, miR-7b). The miR-2 and miR-277 miRNA families are the largest in *Echinococcus*. Taking into account that the sequence outside the seed is divergent among family members and the fact that they have different expression levels, it would be interesting to explore if they act redundantly or some members have acquired new targets and therefore novel functions. We also detected miR-4990 [15] and identified miR-new-1 in both *Echinococcus* species (Additional file 5), which represent at first instance *Echinococcus*-specific miRNA families (Table 4), since they were not detected in any other genus. Both of them were also very recently reported in *E. granulosus s. s.* [37]. Thus, the 37 miRNAs identified in this study represent 30 unique miRNA families. It has been suggested that miR-3479 family is only present in *E. granulosus*, *S. mansoni* and *S. japonicum* [37]. However, in the present study we did not find platyhelminth-specific miRNA families. The analysis of *E. canadensis* G7 miRNA families (Table 4) showed that miR-3479 belongs to miR-92 family that is conserved not only in Platyhelminths but also in Vertebrata, Nematoda, Arthropoda and Annelida.

**Table 4:** **Evolutionary origin of** ***Echinococcus canadensis*** **G7and** ***Echinococcus granulosus sensu stricto*** **G1 miRNA families**

**Evolutionary origin**	**Family name**	***Echinococcus*** **miRNA name**	**Seed**	**Conservation** ^**a**^
Eumetazoa	miR-10	miR-10-5p	ACCCUGU	+/+/+/+/+
Bilateria	let-7	let-7-5p	GAGGUAG	−/+/+/+/+
	miR-1	miR-1-3p	GGAAUGU	−/+/+/+/+
	miR-7	miR-7a-5p	GGAAGAC	−/+/+/+/+
		miR-7b-5p	GGAAGAC	−/+/+/+/+
	miR-8	miR-8-3p	AAUACUG	−/+/+/+/+
	miR-9	miR-9-5p	CUUUGGU	−/+/+/+/+
	miR-22	miR-745-3p	GCUGCCU	−/+/−/+/+
	miR-31	miR-31-5p	GGCAAGA	−/+/+/+/+
	miR-71	miR-71-5p	GAAAGAC	−/−/+/+/+
	miR-92	miR-3479-3p	AUUGCAC	−/+/+/+/+
		miR-new-3-3p	AUUGCAC	−/+/+/+/+
	miR-96	miR-96-5p	UUGGCAC	−/+/+/+/+
	miR-124	miR-124a-3p	AAGGCAC	−/+/+/+/+
		miR-124b-3p	AAGGCAC	−/+/+/+/+
	miR-125	miR-125-5p	CCCUGAG	−/+/+/+/+
	miR-133	miR-133-3p	UGGUCCC	−/+/+/+/+
	miR-153	miR-153-3p	UGCAUAG	−/+/+/+/+
	miR-184	miR-4988-3p	GGACGGA	−/+/+/+/+
	miR-190	miR-190-5p	GAUAUGU	−/+/+/+/+
	miR-219	miR-219-5p	GAUUGUC	−/+/−/+/+
	miR-281	miR-281-3p	GUCAUGG	−/+/+/+/+
Protostomia	bantam	bantam-3p	GAGAUCG	−/−/+/+/+
	miR-2	miR-2a-3p	AUCACAG	−/−/+/+/+
		miR-2b-3p	AUCACAG	−/−/+/+/+
		miR-2c-3p	UCACAG	−/−/+/+/+
	miR-36	miR-36-3p	CACCGGG	−/−/+/+/+
	miR-67	miR-307-3p	CACAACC	−/−/+/+/+
	miR-87	miR-87-3p	UGAGCAA	−/−/+/+/+
	miR-277	miR-277-3p	AAAUGCA	−/−/+/+/+
		miR-4989-3p	AAAUGCA	−/−/+/+/+
		miR-new-2-3p	AAAUGCA	−/−/+/+/+
	miR-279	miR-61-3p	GACUAGA	−/−/+/+/+
	miR-1993	miR-2162-3p	AUUAUGC	−/−/+/+/+
Lophotrochozoa	miR-1992	miR-1992-3p	CAGCAGU	−/−/−/−/+
*Echinococcus*-specific	novel	miR-4990-5p	CUCCUCA	−/−/−/−/−
	novel	miR-new-1-3p	AAUUCGA	−/−/−/−/−

^a^Seed conservation profile is shown. Cnidaria/Vertebrata/Nematoda/Arthrophoda/Annelida.

### Analysis of sequence conservation of *Echinococcus* miRNAs

**Figure 7:**
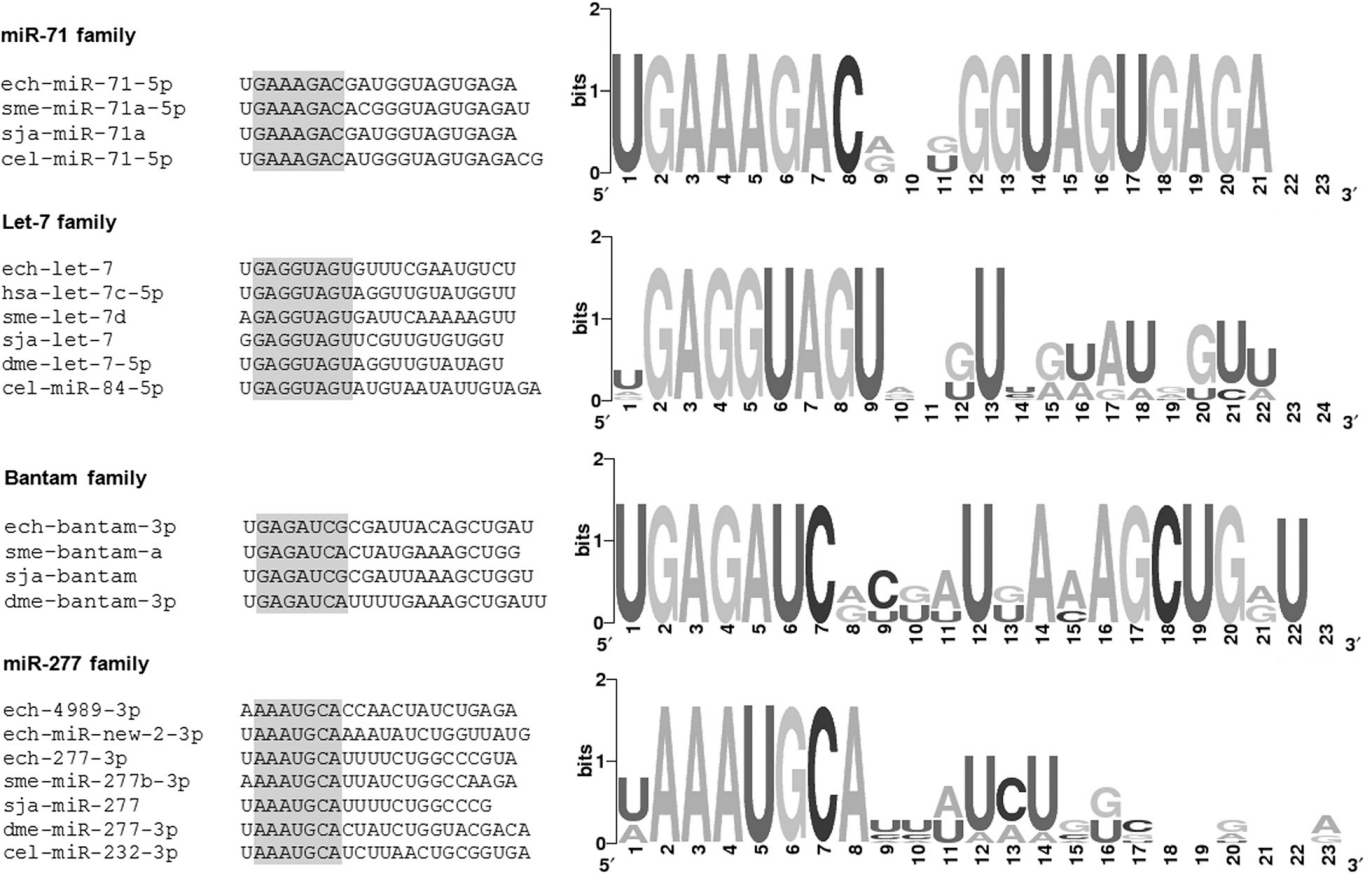
**Sequence alignments and logos of selected** ***Echinococcus canadensis*** **G7 miRNAs.** Highly expressed miRNAs that are absent or divergent from host homologs are shown. ech: *Echinococcus granulosus* s. l*.* sme: *Schmidtea mediterranea.* sja: *Schistosoma japonicum.* dme: *Droshophila melanogaster.* cel: *Caenorhabditis elegans*. hsa: *Homo sapiens.* The seed sequence (nt 2–8) is shadowed in grey color.

To investigate the degree of conservation of those *Echinococcus* miRNAs that belong to conserved miRNA families we compared the full-length mature sequences (using as criteria seed conservation and ≥70% identity) to miRBase v20 entries of three selected model species: *Homo sapiens* (Chordata), *Caenorhabditis elegans* (Nematoda)*, Droshophila melanogaster* (Arthropoda) and two platyhelminths: *Schmidtea mediterranea* (Turbellaria) and *Schistosoma japonicum* (Neodermata). Interestingly, the majority of *Echinococcus* miRNAs 29/35 (83%) have homologs with ≥ 70% identity in the free living *S. mediterranea* (Turbellaria) and only 23/35 (66%) in *S. japonicum*, despite the fact that the latter organism and *Echinococcus* comprise a clade within Neodermata and *S. mediterranea* was found to be basal to this clade [47]. This could be due to specific loss of some miRNAs in the Trematoda lineage, such as the deeply conserved miR-9 and the lophotrochozoan-specific miR-1992 and/or the fact that the *S. japonicum* miRNA repertoire is still incomplete despite the several high-throughput studies that have been done to date for this parasite [43,48,49]. It is worth mentioning that 19/35 (54%) of *Echinococcus* miRNAs have homologs in *Drosophila melanogaster,* while a smaller number 12/35 (34%) and 13/35 (37%) have homologs in *C. elegans* and *H. sapiens*, respectively (Additional file 7). This finding is unexpected since *C. elegans* (Nematoda) and *D. melanogaster* (Arthropoda) belong to the same ecdysozoan clade of protostomes, and *Echinococcus* is more closely related to them than to *Homo sapiens* (Chordata), due to its affiliation to Lophotrochozoa [50]. Additionally, we found that full-length mature sequences of 5 *Echinococcus* miRNAs: miR-1, miR-31, miR-124, miR-125, and miR-190 are highly conserved across all the species analyzed here suggesting further functional conservation. We also found that let-7, miR-new-2 and miR-4989 (miR-277 family) (Figure 7), miR-4988 (miR-184 family) and miR-new-3 (miR-92 family); are highly divergent at the nucleotide level from their homologs even among the analyzed platyhelminths.

## Discussion

*E. canadensis* G7 is a cestode parasite that shows developmental and morphological particularities, and is an important cause of cystic echinococcosis in humans and livestock. However, many aspects of its biology are poorly understood hampering the development of new control strategies. Using a high-throughput approach we have identified and analyzed the expression profile of miRNAs in *E. canadensis* G7. The main aspects of the results obtained are discussed below.

### miRNAs are the preponderant class of small RNA population in *E. canadensis* G7

We have found that miRNAs are the most abundant type of small RNAs in protoscoleces as well as in cyst walls of *E. canadensis* G7. The percentage of miRNAs in PSG7 (53%) is very similar to the previously reported [15] which reached ~45%. This confirms the high level of miRNA expression in the PS stage and probably explains the possibility of identifying several miRNAs by a low scale approach [15]. We observed that miRNAs were also the most expressed type of small RNAs in *E. granulosus* s. s., in coincidence with the results recently obtained [37] for annotated small RNAs in *E. granulosus* s. s.

Small RNAs compatible with piRNAs were not detected in this work. This observation is in agreement with the absence of a canonical PIWI protein in *Echinococcus* genomes [17,51] and with the fact that piRNAs were not identified in any other platyhelminth parasite so far [52]. This is in contrast with findings in the free living flatworm *Schmidtea mediterranea*, where piRNAs were highly expressed in neoblasts [30,53]. Since neoblasts, or somatic totipotent stem cells, also occur in parasitic flatworms such as *E. multilocularis* [52,54] it would be interesting to search for piRNA-like molecules in stem cell-enriched samples of *Echinococcus*. It is also possible that a yet unknown mechanism operates in parasitic flatworms in order to silence transposable elements [52].

In this work, 5’ half-tRNAs from host origin were found in the parasite larval stage interfacing with the intermediate host, i.e. cyst walls. Since small RNA fragments derived from tRNAs have emerged as a novel type of regulatory RNAs able to inhibit translation in response to stress [55], including pathogen-induced stress [56,57] it would be very interesting to analyze the role of these tRNA fragments in the host response to infection.

### A comprehensive repertoire of *E. canadensis* G7 miRNAs

In this work, we obtained a comprehensive miRNA repertoire of *E. canadensis* that includes conserved as well as novel miRNAs. Deep sequencing technology allowed expanding the *E. canadensis* G7 miRNA set to 37 loci. Using a highly stringent annotation protocol, which allowed obtaining high confidence predictions, we identified and confirmed the expression in *E. canadensis* G7 of several miRNAs that were considered lost in *Echinococcus* [36], lack experimental validation [41] or had not been reported in *E. canadensis* G7 so far. Furthermore, by adjusting the parameters of the miRDeep2 algorithm, we could annotate two additional conserved miRNAs, miR-7b and miR-96, which show a high level of expression, particularly in the PS stage. Recently, a higher number of new miRNAs was reported in *E. granulosus* s. s. G1 [37]. We consider that the absence of these miRNAs in the *E. canadensis* G7 miRNA repertoire could be due to the fact that most new miRNAs identified in that work were highly expressed only in adult worms, a stage not analyzed in the present study. Other possible explanation is the highly stringent annotation pipeline used in this work. We cannot rule out that using a reference genome of another species may have contributed to the low number of novel candidate miRNAs identified.

### Few *E. canadensis* G7 miRNAs account for most miRNA expression in the intermediate host

Few miRNAs account for a high percentage of miRNA expression in *E. canadensis* G7 PS and CW. In particular, miR-71 and let-7 were among the most abundant miRNAs in all samples analyzed. High expression of miR-71 was also observed across platyhelminths such as the cestodes *Taenia multiceps* [58] and *Taenia saginata* [59] and the trematode *S. japonicum* [43,60], having this miRNA the highest number of predicted targets in *S. mansoni* [61]. So far, there is no information about the function of miR-71 and let-7 in *Echinococcus*. However, miR-71, a bilaterian miRNA absent in vertebrates, is known to be involved in *C. elegans* lifespan regulation [62] and stress responses [63]. Since the metacestode lives long term in the hostile host environment, probably producing molecules to modulate the host immune response [51] it would be interesting to determine whether miR-71 is involved in responding to the stress induced by the immune system of the host and/or the longevity of the parasite. Regarding the other highly expressed miRNA, let-7, it was also found to be highly expressed in *S. japonicum* eggs and adult worms [43]. Let-7 was one of the first discovered miRNAs and was shown to be essential for temporal development in *C. elegans* [64]. In addition, it has been shown that this miRNA can regulate the mice insulin response by targeting several genes of the insulin-PI3K-mTor pathway, including the insulin receptor [65]. Since *E. multilocularis* metacestode expresses tyrosine kinases of the insulin receptor family [66] which were recently shown to respond to host insulin promoting parasite growth [67], it would be interesting to determine whether *Echinococcus* let-7 targets the insulin pathway genes playing a role in controlling the parasite response to the host hormone.

Two other highly expressed miRNAs were miR-1 and miR-9. Both of them are deeply conserved through evolution with known roles in muscle [68] and neural development [69], respectively. Since muscle and nerve cells are present in PS and CW [70-73] although showing different level of organization and complexity, expression of miR-1 and miR-9 miRNAs is expected in all samples. Taking into account that miR-9 has emerged as an important regulator in development [69] and miR-1 is able to change the whole cellular mRNA profile thus defining cell fate [74], it would be interesting to determine if these miRNAs exert similar functional roles in *Echinococcus* given their high expression level with respect to other miRNAs. Similar biased expression to the observed in this study was reported for different developmental stages of *S. japonicum* with five miRNAs accounting for more than 80% of all miRNA reads [60]. In coincidence with our results, miR-71, let-7 and miR-1 were recently reported among the top 5 most expressed miRNAs in *E. granulosus* s. s. G1 protoscoleces and cysts [37].

### Stage-biased miRNA expression is a feature of *E. canadensis* G7

The high proportion of differentially expressed miRNAs suggests stage-associated functional roles. Although the function of miRNAs in *Echinococcus* biology is so far unknown, differentially expressed miRNAs between PS and CW from *E. canadensis* G7 could be involved in post transcriptional regulation of particular sets of protein coding genes that were found to be differentially expressed in each stage [22,51] and thus, in maintaining protoscolex and cyst wall features. Several differentiation and reorganization events must occur in the germinal layer of the metacestode in order to develop to the PS stage. Germinal layer is composed by tegumental, muscle; glycogen/lipid storing, duct and flame cells [73], nerve cells [72] as well as undifferentiated germinative stem cells, which give rise to brood capsules that in turn, develop PS. The protoscolex has several distinctive features, such as a rostellum with hooks, suckers, and the ability to exert coordinated movements. Muscular and nervous systems are present in both stages but they show further organization and complexity in the protoscolex [71], with serotoninergic [70,72] and acetylcholinergic nervous systems [72] only present at the later stage. In addition, differences in metabolism were also described, with the germinal layer appearing to possess a higher metabolic activity in order to count with energy and intermediate metabolites for the synthesis of the laminated layer toward the outside of the cyst and the generation of brood capsules containing PS toward the inside [22].

miR-277 and miR-4989 are two up-regulated miRNAs in CW samples that belong to the same family (miR-277). In *Drosophila* spp., it was shown that miR-277 targets genes involved in branched-chain amino acid catabolism and activates TOR, which regulates cell growth and metabolism in response to environmental cues [75]. It would be interesting to determine if these two up-regulated miRNAs in CW samples play similar roles in *Echinococcus* thereby promoting germinal layer continuous regeneration. miR-277 also showed differential expression during *S. mansoni* development [43]. Among the up-regulated miRNAs in PS samples are miR-7a and miR-7b (miR-7 family). A recent study of the function of miR-7 in the model organism *Drosophila* revealed that miR-7 regulates *Drosophila* wing growth by controlling cell cycle phasing and cell mass [76]. As mentioned above for miR-277, it would be interesting to determine if miR-7 family members have a developmental role in *Echinococcus* parasites.

Although the conserved miRNA repertoire of *E. canadensis* is very similar to that of *E. granulosus* s. s. [37], miRNA differential expression profiles highly differ between these two species. For example, miR-4989 and miR-277, two miRNAs that are organized in a cluster and share the seed region, are among the up-regulated miRNAs in *E. canadensis* CW while they show down regulation in *E. granulosus* s. s. CW with respect to PS [37]. Since one of the most important differences among *E. granulosus* s. l. species is intermediate host preference, and CW interfaces with the intermediate host, we regard that divergent miRNA profiles in this stage can contribute to differences in intermediate host specificity.

### Most *E. canadensis* G7 miRNA families are evolutionary conserved

The small number of novel families found here is in contrast to that reported in the free living flatworm *S. mediterranea* [30] where 34 conserved miRNA families and 45 novel ones were found. In addition to secondary loss of conserved miRNA families and the few novel miRNAs found in *E. canadensis*, a lower number of members in some families with respect to other flatworms was observed. For these reasons the miRNA complement of this parasite is smaller (37 precursor sequences identified in this study) than that of the free-living flatworm *S. mediterranea* (148 precursor sequences, miRBase 20). Although it has been suggested that secondary loss is rare and mature miRNAs are under intense negative selection [77,78], a remarkable loss of conserved miRNA families: 14 bilaterian, 4 protostomian and 1 lophotrochozoan was confirmed in this work for *Echinococcus* regarding that 46 conserved miRNA families are expected for a lophotrochozoan organism [36,78]. We hypothesize that the lower rate of acquisition of novel miRNAs and the remarkable loss of conserved miRNAs could be related to a parasitic lifestyle and reduced morphological complexity. Since extreme losses of genes and pathways were found in *Echinococcus* with respect to free living platyhelminths [17], a reduced number of miRNAs is expected. Additionally, it has been suggested that expansions of the miRNA repertoire appear to be associated with major body-plan innovations during animal evolution [79] and in the same way, miRNA losses to reduced morphological complexity [80].

A recent analysis of the miRNA repertoire in *E. granulosus* s. s. further confirmed the reduced number of conserved miRNA families in *Echinococcu*s [14]*.* In the present study only 5 novel candidate miRNAs were detected for *E. canadensis*. It would be interesting to analyze the miRNA repertoire of other stages, such as adult worms, in order to determine if novel miRNA families are expressed as reported for *Haemonchus contortus* and *E. granulosus* s. s. adult stages [28,37] that were shown to be enriched in novel miRNAs with respect to larval stages. In addition, it would be important to perform the miRNA prediction analysis using *E. canadensis* genome when available.

### Several *Echinococcus* miRNAs are absent or highly divergent from vertebrate host homologs

Several *Echinococcus* miRNAs are absent from vertebrate hosts since they have a protostomian or a lophotrochozoan origin or because they are *Echinococcus-*specific. Although many conserved *Echinococcus* miRNAs belong to miRNA families that are present in the subphylum Vertebrata, many of them are only poorly conserved. The divergence of these *Echinococcus* miRNA sequences found at the nucleotide level with respect to those from other organisms, including other platyhelminths, may likely indicate an accelerated evolution of these miRNAs in the *Echinococcus* lineage. This could imply specific roles for these miRNAs in development, survival and/or host-parasite interaction. Also, this may reflect the more complex life cycles of parasitic species and their ability to adapt to different environments. Interestingly, a recent study reported the detection of *S. mansoni* miRNAs, such as miR-277 and bantam, in host serum and highlighted their diagnosis potential in schistosomiasis [81], specially taking into account that they are not present in the host. In *Echinococcus*, highly expressed miRNAs that are absent from vertebrate hosts, such as miR-71, miR-4989 (miR-277 family) and bantam, or that are divergent from host homolog miRNAs (Figure 7), for example let-7, could be evaluated as candidate targets for diagnosis or intervention strategies.

## Conclusions

The sanitary importance of *E. canadensis* and its particular developmental features highlight the significance of characterizing molecules, such as miRNAs, that are widely recognized as key players in development. The recently generated genomes for *Echinococcus*, together with the improvement of high throughput technologies and available algorithms for miRNA discovery allowed us the identification of additional miRNAs from high-throughput data, thereby expanding *E. canadensis* miRNA repertoire. Using this approach, we performed the first in-depth small RNA profiling of the zoonotic parasite *E. canadensis* G7. We found that miRNAs are the preponderant small RNA silencing molecules in *E. canadensis* G7 suggesting that these small RNAs could be an essential mechanism of gene regulation in *Echinococcus*. We found that some miRNAs are abundantly expressed in all the stages/species analyzed in this study, suggesting that they could be essential in *Echinococcus* larval stages for survival in the intermediate host. Differential expression analysis showed highly regulated miRNAs between life cycle stages of *E. canadensis* G7. Although the function/s of miRNAs in *Echinococcus* is so far unknown, this result suggests that miRNAs could have stage-specific functional roles and/or regulate developmental timing. Here we confirmed the remarkable loss of conserved miRNA families in these cestodes, reflecting their low morphological complexity and high adaptation to parasitism. Furthermore, we identified both parasite specific and divergent miRNAs which are potential biomarkers of infection. This study provides valuable information to understand the complex biology of *Echinococcus* parasites and could help to find new control strategies for the worldwide distributed and mostly neglected diseases they produce.

## Additional files

Additional file 1:
**Primers sequences and efficiencies of all primer pairs used in this study.** This file contains all forward and the reverse sequences and efficiencies of all primer pairs used in this study. Additional file 2:
**Percentages of reads matching ncRNAs and repeats.** This file contains the numbers and percentages of reads matching ncRNAs and repeats in *Echinococcus canadensis* G7 and *Echinococcus granulosus sensu stricto* G1. datasets. Additional file 3:
**Top five most abundant 31 nt sequences not-mapping to** ***Echinococcus*** **genome.** This file contains the sequences of tRNA halves from host origin. Additional file 4:
**Expression level of selected miRNAs measured by RT-qPCR relative to miR-71 in each** ***Echinococcus canadensis*** **G7 stage.** This file contains a supplemental figure that shows the miRNA expression profile of fifteen randomly selected miRNAs relative to miR-71 in CWG7 and PSG7 samples measured by RT-qPCR. Additional file 5:
**Expression profile of the full repertoire of** ***Echinococcus canadensis*** **G7 miRNAs identified in this study.** This file contains mature sequences and normalized read counts of full repertoire of *Echinococcus canadensis* G7 and *Echinococcus granulosus sensu stricto* G1 miRNAs. Additional file 6:
**Precursor sequences and raw read numbers of the full repertoire of** ***Echinococcus canadensis*** **G7 and** ***Echinococcus granulosus sensu stricto*** **G1 miRNAs identified in this study.** This file contains precursor sequences and raw read counts of full repertoire of *Echinococcus canadensis* G7 and *Echinococcus granulosus sensu stricto* G1 miRNAs. Additional file 7:
**Conservation of** ***Echinococcus*** **miRNAs.** This file contains a supplemental figure that shows percentage of miRNAs in *Echinococcus* with homologs in Sme: *Schmidtea mediterranea.* Sja: *Schistosoma japonicum.* Dme: *Droshophila melanogaster.* Cel: *Caenorhabditis elegans*. Hsa: *Homo sapiens*.
